# Effect of Cigarette Smoke on Gut Microbiota: State of Knowledge

**DOI:** 10.3389/fphys.2021.673341

**Published:** 2021-06-17

**Authors:** Xiaohua Gui, Zhongli Yang, Ming D. Li

**Affiliations:** ^1^State Key Laboratory for Diagnosis and Treatment of Infectious Diseases, National Clinical Research Center for Infectious Diseases, Collaborative Innovation Center for Diagnosis and Treatment of Infectious Diseases, The First Affiliated Hospital, Zhejiang University School of Medicine, Hangzhou, China; ^2^Research Center for Air Pollution and Health, Zhejiang University, Hangzhou, China

**Keywords:** cigarette smoking, lung–gut axis, skin-gut axis, intestinal microbiota dysbiosis, systemic disorders

## Abstract

Cigarette smoke is a representative source of toxic chemical exposures to humans, and the adverse consequences of cigarette smoking are mediated by its effect on both neuronal and immune–inflammatory systems. Cigarette smoking also is a major risk factor for intestinal disorders, such as Crohn’s disease and peptic ulcer. On the other hand, cigarette smoking is protective against developing ulcerative colitis. The effects of cigarette smoking on intestinal disorders include changes in intestinal irrigation and microbiome, increases in permeability of the mucosa, and impaired mucosal immune responses. However, the underlying mechanism linking cigarette smoking with intestinal microbiota dysbiosis is largely unknown. In this communication, we first review the current knowledge about the mechanistic interaction between cigarette smoke and intestinal microbiota dysbiosis, which include the likely actions of nicotine, aldehydes, polycyclic aromatic hydrocarbons, heavy metals, volatile organic compounds and toxic gases, and then reveal the potential mechanisms of the lung–gut cross talk and skin-gut cross talk in regulating the balance of intestinal microbiota and the interrelation of intestinal microbiota dysbiosis and systemic disorders.

## Introduction

Cigarette smoking is a leading cause of preventable deaths worldwide. Despite public awareness of the harmful effects of cigarette smoking, it is a global pandemic, with 1.1 billion people being current smokers worldwide (Huxley and Woodward, 2011). Although the prevalence of smoking is declining in developed counties, it is increasing in many developing counties, especially in Asia (Rom et al., 2013). About half of smokers will develop serious smoking-related diseases, such as chronic obstructive pulmonary disease (COPD), cardiovascular disease, and cancers (Stämpfli and Anderson, 2009; Brusselle et al., 2011; Huxley and Woodward, 2011; Csordas and Bernhard, 2013). In addition, second-hand smoke exposure increases the risk of infection by pathogens and causes worsening of other lung diseases, such as asthma (Öberg et al., 2011). Furthermore, cigarette smoking plays a dichotomous role in the development of inflammatory bowel disease (IBD). It worsens the development of Crohn’s disease (CD), but it improves the symptoms of ulcerative colitis (UC) (Opstelten et al., 2016). The toxic components in cigarette smoke have been considered the greatest contributor to serious diseases, and the underlying pathological mechanisms have been widely studied (Hoffmann et al., 2001; Smith et al., 2002). However, these mechanisms are unclearly understood.

There are more than 1 × 10^14^ microbial cells in the human intestine, which play important roles in physicochemical and physiological functions via their dynamic interaction with the host, including intestinal development, barrier integrity and function, metabolism, immunity, inflammation, and neurological signaling regulation (Backhed et al., 2005; Hooper et al., 2012; Nicholson et al., 2012; Braniste et al., 2014). Accumulating evidence from human and animal studies supports that the intestinal microbiome is as important as genetic factors in affecting intestinal and extra-intestinal diseases, such as irritable bowel syndrome (IBS), IBD (Maslowski et al., 2009), cardiovascular disease (Bogiatzi et al., 2018), obesity (Turnbaugh et al., 2006), rheumatoid arthritis (Scher et al., 2020), systemic lupus erythematous (Luo et al., 2018), central nervous systemic (CNS) diseases (De Angelis et al., 2015), and cancers as well (Zhang et al., 2019). The balance of the gut microbiota is highly dynamic, being disturbed by many factors, such as genetics, age, circadian rhythm, dietary habits, antibiotics, and other environmental factors (such as cigarette smoke) (Bennet et al., 2002; Hufeldt et al., 2010; Cho et al., 2012; Yatsunenko et al., 2012; Foster and McVey Neufeld, 2013). At present, considerable experimental work aimed at uncovering mechanistic factors acting between cigarette smoke and intestinal disorders has been circumstantial and ambiguous. In this review, we summarize recent studies exploring the detrimental effect of cigarette smoke on the intestinal microflora, as well as the effect of the lung–gut cross talk and skin-gut cross talk on intestinal microbiota dysbiosis and the interrelation between intestinal microbiota dysbiosis and systemic disorders.

## Effect of Cigarette Smoke Toxicants on Intestinal Microbiota

Cigarette smoke is a complex chemical mixture, including nicotine, aldehydes, polycyclic aromatic hydrocarbons (PAHs), nitrosamines, heavy metals, etc., which is inhaled into the lungs as aerosol particles or free in a gaseous state (Ghio et al., 2008; Centers for Disease Control and Prevention [CDC], 2010, Theron et al., 2012; Lee et al., 2018). These toxic compounds *in vivo* can decrease endogenous antioxidants, increase lipid peroxidation and oxidative stress, and elevate pro-inflammatory factor concentrations in the blood of the host (van der Vaart et al., 2004; Yoshida et al., 2009; Roy et al., 2010). In addition, the toxicants of cigarette smoke swallowed into the gastrointestinal tract induce gastrointestinal microbiota dysbiosis via different mechanisms, such as antimicrobial activity and regulation of the intestinal microenvironment (Bueno et al., 2019). The effects of toxic chemicals in cigarette smoke on the intestinal microbiota are shown in Table 1.

**TABLE 1:** The effects of cigarette smoke toxicants on intestinal microbiota.

**Type**	**Pollutants**	**Content (μg/cigarette)**	**Specie**	**Gut microbiota**	**References**
Nicotine	Nicotine	2500–5000	Human	Bacteroidetes↓ Firmicutes↑ Proteobacteria↑	Lee et al., 2018; Centers for Disease Control and Prevention [CDC], 2010
			Mouse	*Turicibacteraceae↑ Peptococcaceae↑*	Chi et al., 2017a
Polycyclic aromatic hydrocarbons	Benzo[*a*]pyrene	13.4	Mouse	*Bacillus↑ Acinetobacter↑ Erysipelotrichaceae↑ Comamonadaceae↑*	Centers for Disease Control and Prevention [CDC], 2010; Ribière et al., 2016
Volatile organic compounds	Benzene	4–60	Mice	*Helicobacter↑*	Centers for Disease Control and Prevention [CDC], 2010; Sun et al., 2020
Aldehydes	Formaldehyde	0.002–0.05	Mouse	*Turicibacter↓*	Guo et al., 2018; Centers for Disease Control and Prevention [CDC], 2010
	Acrolein	0.0025–0.06	Mouse	Firmicutes↑ Bacteroidetes↓	Centers for Disease Control and Prevention [CDC], 2010; Rom et al., 2017
	Acetaldehyde	0.03–0.65	Mouse	*Bacillus↑ Acinetobacter↑ Erysipelotrichaceae↑ Comamonadaceae↑*	Centers for Disease Control and Prevention [CDC], 2010; Salaspuro, 2003
Toxic Gases	Carbon Monoxide	20000	Mice	*Escherichia coli↓ Salmonella typhimurium↓ Enterococcus faecalis↓*	Onyiah et al., 2013; Centers for Disease Control and Prevention [CDC], 2010
	Hydrogen Sulphide	85	Pigs	Firmicutes↑ Proteobacteria↑ Bacteroides↓	Cui et al., 2020; Centers for Disease Control and Prevention [CDC], 2010
Heavy Metals	Cadmium	0.0016–0.101	Mouse	Lachnospiraceae↓ Lactobacillaceae↓ Turicibacter↑ Coprococcus↑ Streptococci↑	Breton et al., 2013a; Centers for Disease Control and Prevention [CDC], 2010
			Mouse	Firmicutes↑ Proteobacteria↑ Bacteroidetes↓	Jin et al., 2017
	Lead	0.006–0.149	Mouse	Bacteroidetes↓ Firmicutes	Centers for Disease Control and Prevention [CDC], 2010; Wu et al., 2016
			Mouse	Bacteroidetes↓ Firmicutes↑ Proteobacteria↑	Xia et al. (2018)
	Arsenic	0.0015–0.021	Mouse	Bacteroidetes↓ Firmicutes↑	Guo et al., 2014; Centers for Disease Control and Prevention [CDC], 2010
			Mouse	Firmicutes↑ Verrucomicrobia↓	Chi et al., 2017b

### Effect of Nicotine on Intestinal Microbiota

Nicotine, as the primary active substance of tobacco, is inhaled into the lungs and fast absorbed in the pulmonary alveoli and is also absorbed through the skin and gastrointestinal tract. Nicotine can exert multiple favorable and unfavorable physiological functions *in vivo*, such as increasing of metabolic rate, suppressing appetite, regulating body weight, influencing neural activities, alleviating UC, and aggravating the symptoms of CD (Chu et al., 2013; Rupprecht et al., 2016; Chi et al., 2017a; Hu et al., 2018). Accumulating evidence from animal and human studies supports the view that cigarette smoking affects the composition of the intestinal microbiota (Wang et al., 2012; Kobayashi and Fujiwara, 2013). For instance, Lee et al. (2018) reported that cigarette smoking increases the phylum of Bacteroidetes and decreases the phylum of Firmicutes and Proteobacteria in current smokers compared with never smokers. In a rat model, cigarette smoking significantly decreases the concentrations of several organic acids, such as acetic acid, propionic acid, butyric acid, and valeric acid, and the population of *Bifidobacterium* in the cecum (Tomoda et al., 2011). Exposure to cigarette smoke elevates the intestinal pH, which possibly benefits some bacteria, enabling them to thrive and cause intestinal microbiota dysbiosis. Chi et al. (2017a) reported that *Turicibacteraceae* and *Peptococcaceae* significantly increased in male mice when the test group was treated with nicotine through drinking water at a concentration of 60 mg/L for 13 weeks and further found that there was a significant difference between male and female mice. Dysbiosis of the intestinal microbiota may contribute to the pathogenesis of IBD (Thompson-Chagoyán et al., 2005), and patients with IBD have greater adherent bacteria numbers than healthy persons (Swidsinski et al., 2002). As known, cigarette smoking can alleviate UC, whereas it worsens the symptoms of CD. Although the effect of cigarette smoking is unequal to that of nicotine itself, the clinical evidence suggests that nicotine or its metabolites are responsible for the health effect of cigarette smoking in patients with UC. Indeed, the clinical value of nicotine is limited because of its multiple negative effects, including addiction, headache, and nausea. The information on the influence of nicotine on intestinal microbiota is partly observational, a great deal of research work thus is needed to explore the mechanism of nicotine-induced dysbiosis of the intestinal microbiome.

### Effect of Polycyclic Aromatic Hydrocarbons on Intestinal Microbiota

Polycyclic aromatic hydrocarbons are a large group of chemicals with two to seven fused aromatic rings, including benzo[*a*]pyrene (B[*a*]P), benzopyrene, coronene, anthanthrene, and perylene among others. These compounds are formed mainly as result of the thermal cracking of organic resources and incomplete burning of organic material at low temperature. They enter the human body through ingestion of contaminated food and water and inhalation of air polluted by cigarette smoke, automobile exhaust, and industrial waste gas (Diggs et al., 2011). PAHs are also swallowed during cigarette smoking. They induce diverse diseases (such as diarrhea, asthma, and cancer) because of their toxicity, mutagenicity, and carcinogenicity. A recent study reported that intestinal microbiota can transform xenobiotic compounds into non-hazardous or less toxic substances through fermentation, which implies that detoxification of PAHs may be carried out in the gastrointestinal tract through the co-action of multiple enzymes (Nogacka et al., 2019). Van de Wiele et al. (2005) reported that naphthalene, phenanthrene, pyrene, and B[*a*]P can be converted into estrogenic metabolites by fecal microflora in a gastrointestinal simulator. In a murine model, excess B[*a*]P ingestion significantly alters the diversity and abundance of the intestinal microbiota, causes moderate inflammation in ileal and colonic mucosa, and increases the penetrability of the ileal segment (Ribière et al., 2016). From the opposite point of view, Defois et al. (2017) reported that B[*a*]P exposure did not significantly alter the structure of the intestinal microbiome in an *in vitro* assay. Although murine models are widely used in intestinal microbiota studies, there are significant differences between murine and human intestinal microbiota. In addition, the doses of B[*a*]P used obviously are higher than that of the daily living environment, and the alteration of the intestinal microbiota in response to B[*a*] P exposure is not equal to that of a restricted microbial composition. To date, there is little information on the effect of human exposure to individual PAHs on the intestinal microbiome except for some contact with B[*a*]P. Also, PAH is a complex chemical compound, and several individual PAHs may influence the composition of the intestinal microbiota synergistically, and the ratio of individual PAHs may affect the results. Therefore, a systematic study of the effect of PAHs on the composition of the intestinal microbiota is greatly needed.

### Effect of Volatile Organic Compounds on Intestinal Microbiota

Cigarette smoke contains large amounts of toxic volatile organic compounds (VOCs), such as isoprene, acrylonitrile, 1, 3-butadiene, acrylonitrile, styrene and benzene, which are inhaled into respiratory tract and induce different diseases, such as respiratory and cardiovascular diseases, and cancers (Pazo et al., 2016). However, the studies reported the effect of VOCs on intestinal microbiota are rather limited. Sun et al. (2020) reported that C57BL/6 mice were exposed to different levels of benzene by subcutaneous injection for 30 days and found that the overall structure of the intestinal microbiome was altered. Exposed to 150 mg/kg benzene, the phylum of Actinobacteria and the genus of *Helicobacter* were significantly increased in the cecal contents and feces of mice (Sun et al., 2020). Benzene exposure suppresses the components and functions of immune system of the hosts (Kirkeleit et al., 2006; Bahadar et al., 2014). However, the underlying mechanisms of benzene exposure-related intestinal microbiota dysbiosis is unclear. Current research primarily focuses on identifying and evaluating the impact of toxicants of volatile organic compounds on public health (Mögel et al., 2011; Kim et al., 2018). Whether the toxicants attribute to the dysbiosis of intestinal microbiota and whether the toxicants alter intestinal microbiota through sole and/or multiple mechanisms remains to be further determined.

### Effect of Aldehydes on Intestinal Microbiota

Aldehydes, such as saturated (acetaldehyde) and unsaturated (acrolein and croton-aldehyde) compounds, are extremely toxic owing to their high reactivity and induce various disorders and diseases in humans and animals (Colombo et al., 2009; Fuchs et al., 2010; Cho et al., 2017). Cigarette smoke as one of the major sources of aldehydes exposure contains various aldehyde chemicals, and some aldehydes are swallowed into the gastrointestinal tract as particles. The concentrations of acrolein and other aldehydes in saliva and exhaled breath condensate of heavy smokers are about 10-fold higher than those of healthy persons (Colombo et al., 2009). In addition, endogenous aldehydes are produced during the metabolism of nutrient substances and the biotransformation of environmental agents and drugs (Voulgaridou et al., 2011). Exposure to aldehydes has been associated with different diseases, such as atherosclerosis and hypertension, which accompany intestinal microbiota dysbiosis (Srivastava et al., 2011; Yoshida et al., 2011; Brandsma et al., 2017), see Table 1.

Acrolein, a sample of a completely characterized unsaturated aldehyde, is an environmental and food contaminant and a lipid-derived endogenous metabolite and exhibits inhibitory activity against a broad range of gram-positive and gram-negative bacteria, yeasts, molds, and protozoa (Engels et al., 2016; Chen et al., 2017). Acrolein-fed mice show a significant increase in Firmicutes and a decrease in the Bacteroidetes phylum compared with the control group (Rom et al., 2017). Vollenweider et al. (2010) revealed that acrolein increases the oxidative stress of bacteria through reaction with free thiol groups, ultimately leading to cell death. Moreover, acrolein modifies the DNA-binding domain of nuclear factor-κB (NF-κB) to regulate the expression of the antioxidant defense gene; as a consequence, it causes immunosuppression. Lambert et al. (2005) reported that acrolein had a significant effect on inhibiting the binding of NF-κB1 to the interleukin (IL)-2 promoter. In addition, acrolein adduct to reactive cysteine residues in the active center of function proteases to inhibit their activation such as tyrosine phosphatases and caspases, which interrupts the normal cellular signaling cascades and induces necrotic and apoptotic cell death (Finkelstein et al., 2001; Takeuchi et al., 2001; Stevens and Maier, 2008).

Acetaldehyde, a low-molecular-weight aldehyde, has high reactivity and induces different diseases, such as liver injury and gastrointestinal cancers. Many intestinal bacteria can convert acetaldehyde into ethanol and gain energy through fermentation, which leads to the overgrowth of relevant bacteria (Salaspuro, 2003). Moreover, acetaldehyde increases the permeability of intestinal tract by reducing the expression of tight-junction and adherent- junction proteins of intestinal mucosa. Microorganisms and endotoxin cross the intestinal mucosal barrier into the blood and induce endotoxemia, subsequently leading to injury to the liver and other organs, intestinal inflammation, and rectal carcinogenesis (Elamin et al., 2013). In addition, acetaldehyde and reactive oxygen species induces Kupffer cells to release reactive oxygen species, proinflammatory cytokines, and chemokines that lead to neutrophil infiltration (Ceni et al., 2014). Neutrophils reaching the intestinal epithelium regulate the intestinal inflammatory responses through release of tissue-damaging compounds, which cause translocation of intestinal microbiota (Gagliani et al., 2014; Starling, 2017). However, most reported studies were focused only on aldehyde *per se*, whereas exposure to the mixtures of aldehydes appears to be more common than that to a single aldehyde. Therefore, further research is warranted to elucidate the mechanisms underlying the effects of aldehyde mixtures on the intestinal microbiota.

### Effect of Toxic Gases on Intestinal Microbiota

The main toxic gases of tobacco smoke, including carbon dioxide, carbon monoxide (CO), hydrogen sulfide (H_2_S), ammonia, hydrogen, and nitrogen oxides (NO_*x*_), are inhaled into lung and entered into blood through alveolar exchange, which affects the O_2_ transport, decreases the pH of blood, and induces systemic inflammation and diseases (Sloan et al., 2010; Guais et al., 2011; Ortiz and Grando, 2012). Approximately 20–30 mls of CO are inhaled from each cigarette and convert about 15% of hemoglobin into carboxyhemoglobin, which affects the O_2_ transport and results in systemic hypoxia (Sloan et al., 2010). Indeed, the amount of CO in tobacco smoke is too small to lead to hypoxia and the body can produce increased numbers of red blood cells to compensate (West, 2017). Exogenous CO administration at low concentrations is protective against intestinal inflammation (Ryter and Choi, 2007; Takagi et al., 2015). Onyiah et al. (2013) reported that CO exposure altered the intestinal microbiome and induced bacterial species that express HO homologs involved in iron acquisition. CO from cigarette smoke might alleviate the clinic symptoms of patients with IBD through regulating the balance of intestinal microbiota.

Hydrogen sulfide as another major constituent of cigarette smoke causes both pro-inflammatory and anti-inflammatory responses, even induce metabolic disorders in a few vertebrates (Olson, 2011). In the dextran sulfate sodium-induced colitis model, H_2_S donors (diallyl disulfide) promotes biofilm formation and reduces growth of planktonic bacteria (Motta et al., 2015). After exposed to different levels of H_2_S (0, 5, 10, and 15 mg/m^3^) for 28 days, the abundance of Firmicutes and Proteobacteria were increased and the abundance of Bacteroides were decreased in the weaning pigs (Cui et al., 2020). However, the influences of toxic gasses of cigarette smoke on intestinal microbiome depend on multiple factors, such as the production location of tobacco, the processing of cigarette, the state of incomplete combustion and cigarette smoked indoor or outdoor.

### Effect of Heavy Metals on Intestinal Microbiota

Cigarette smoke contains many heavy metals (such as cadmium, arsenic, lead, chromium, iron, mercury, nickel, and vanadium), which are inhaled into the lungs as aerosol particles or free in a gaseous state (Chiba and Masironi, 1992). Some heavy metals are transferred to the gastrointestinal tract during deglutition, and a significant fraction of heavy metals is ingested (Satarug et al., 2003). Heavy metal exposure could cause intestinal microbiota dysbiosis, indicating that the study of heavy metal exposure is a new topic to explore in working out the pathogenic mechanisms of cigarette smoke. After mice were given drinking water containing cadmium 100 mg/L for 8 weeks, Breton et al. (2013a) found significant differences in the relative abundance of Lachnospiraceae, Lactobacillaceae, and Erysipelotrichaceae in both cecal and fecal samples. Jin et al. (2017) revealed that a low dose of cadmium decreased the relative abundance of Firmicutes and Proteobacteria but increased the relative abundance of Bacteroidetes in the cecum and feces of male mice. Adult male rats exposed to lead (32 mg/L in drinking water) for 40 weeks had a decreased ratio of Bacteroidetes/Firmicutes and increased abundance of Desulfovibrionaceae, *Barnesiella*, and *Clostridioides* XIVb (Wu et al., 2016). Exposure to arsenic (3 mg/L in drinking water) for 90 days increased the abundance of Firmicutes but decreased the abundance of Bacteroidetes in the intestines of mice (Guo et al., 2014). Heavy metals exposure thus is linked to intestinal microbiota dysbiosis, whereas the distinction of intestinal microbiota might contribute to the species and dose of heavy metals. Previous studies have demonstrated that the heavy metal species has a specific effect on the transport, oxidative, and inflammatory status of the gut epithelium (Breton et al., 2013b; Guo et al., 2015). Interestingly, different types of dysbiosis are likely related to various diseases, including inside and outside the intestine. Several intestinal microorganisms might interact with heavy metals via active or passive absorption (Halttunen et al., 2008; Kinoshita et al., 2013). As mentioned above, the effect of heavy metal on the dysbiosis of the intestinal microbiota depends on various factors, including heavy metal species and toxicity, dose effect, chronic or acute exposure, and absorption pattern.

## Potential Mechanisms of Organ Cross-Talk

### Potential Mechanisms of the Lung-Gut Axis

**FIGURE 1:**
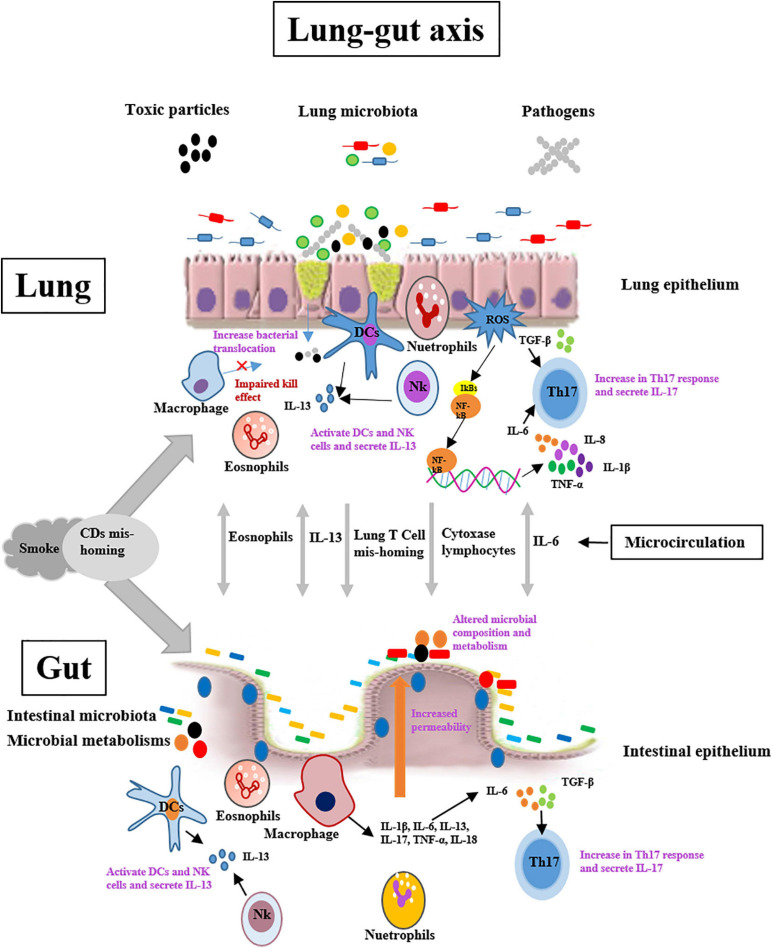
Potential mechanisms of lung-gut cross talk include lung-originating T-cell and eosinophils mis-homing and increases the expression of cytochrome oxidase in lymphocytes. Cigarette smoke exposure may exert important roles in organ cross-talk by affecting these processes, and/or by causing DCs mis-homing in the lungs and gut. Systemic IL-6 combined with local TGF-β may drive cross-organ Th17-polarized inflammation. Systemic IL-13 may stimulate NK cell and macrophage responses in organ cross-talk. DCs = dendritic cells; IL-6 = Interleukin-6; TGF-β = Transforming growth factor-β; NK cell = Natural killer cell.

Cigarette smoking is the primary causes of COPD and about 80% present patients with COPD are the former or present smokers (Barendregt et al., 1997). The epidemiology has demonstrated that patients with chronic lung diseases have a higher prevalence of intestinal diseases, such IBD and IBS (Roussos et al., 2003; Rutten et al., 2014). Moreover, pulmonary and intestinal diseases demonstrate many overlapping changes, such as the common risk factors, mucus reduction, increase of permeability, and low expression of tight-junction proteins. In addition, respiratory and gastrointestinal epithelia have structural similarities, having the same embryonic origin (Budden et al., 2016). These clinical studies indicated that the pulmonary disorders may be implicated in intestinal diseases through the underlying mechanisms, such as the lung-gut axis. However, the mechanism of lung-gut axis is ambiguous. It is likely that the underlying mechanism is multifactorial, as are COPD and IBD (Figure 1).

Smoke exposure increases the number of lung dendritic cells (DCs), which may reduce the number of myeloid DCs migrating to the lymph nodes (Lommatzsch et al., 2010). In addition, the current paradigm of T-cell homing to the intestinal tract associates with the induction of α4β7 and C chemokine receptor 9 by Peyer’s patches and mesenteric lymph node DCs (Ruane et al., 2013). Smoke inhalation also results in the accumulation of DCs in the intestinal Peyer’s patches of wild-type mice (Verschuere et al., 2011). The accumulation of CD4 + T cells correlates positively with the increase in DCs and the apoptosis of cells over-lying the epithelial tissue of the intestine. Unexpectedly, a recent study reported that lung DCs induced intestinal immunity responses against a highly pathogenic strain of *Salmonella* via upregulating the gut-homing integrin α4β7 and inducing T-cell migration to the intestinal tract (Ruane et al., 2013). During respiratory tract inflammation, the bronchus lymphoid tissue mediates the lymphocyte trafficking from the lung tissue through the microcirculation (Keely et al., 2012). However, the change in the homing properties of pulmonary lymphocytes is likely to contribute to the pathogenesis of other organs associated with COPD patients, such as rheumatoid synovium (Manolios et al., 1991; Ainslie et al., 2002). Whether this phenomenon contributes to IBD in patients with COPD is unclear. Moreover, an increase in cytochrome oxidase (CytOx) activity in peripheral lymphocytes of COPD patients accompanies the increase of CytOx activity in wasting skeletal muscle (Polverino et al., 2009). A similar result also is observed in other chronic inflammatory diseases, including rheumatoid arthritis and asthma, but whether the same event occurs in IBD is uncertain (Sauleda et al., 2000). In addition, allergen challenge to the lungs stimulated not only the pulmonary eosinophilic inflammation, but also increase the number eosinophils in intestinal tract, and vice versa (Olbrich et al., 2020).

Interleukin-6 (IL-6) exerts a pivotal role in regulating many cellular process and has been implicated in the pathogenesis of various inflammatory disorders such as COPD and IBD (Mudter and Neurath, 2007; Danese and Gao, 2010; Ruwanpura et al., 2011). The systemic concentration of IL-6 is elevated in patients with lung disorders, and IL-6 is associated with the apoptosis of cells in pulmonary tissue (Ruwanpura et al., 2011). Moreover, IL-6 combined with transforming growth factor beta (TGF-β) is an important trigger in the development of the Th17 subset of T-helper cells (Tulic et al., 2016). These Th17 cells secret various cytokines (such as IL-17, IL-21, IL-22, IL-26, and tumor necrosis factor-α) that regulate host immune responses against mucosal disorders (Le Rouzic et al., 2017). Also, IL-6 and Th17 cells are associated with IBD, and IL-6 and Th17 cell-related cytokines have been detected in blood and the inflammatory mucosa of the intestinal tract in these patients (Keely et al., 2012). Accumulating evidence demonstrates that TGF-β plays an important role in the mucosal remodeling in chronic pulmonary and intestinal inflammation (Kokturk et al., 2003; Ihara et al., 2017). It is likely that the combination of IL-6 and TGF-β causes the development of an extra-organ Th17-polarized immune response. In addition, IL-6 induces the overexpression and nuclear translocation of transcription factor STAT-3 through *trans-*signaling, which results in T-cell resistance to apoptosis by inducing the expression of the anti-apoptotic factors bcl-2 and bcl-xl. Thus, T-cell accumulation causes chronic intestinal inflammation that can be treated with anti-IL-6 receptor antibodies (Mudter and Neurath, 2007). Although anti-IL-6 receptor antibodies are effective in the therapy of some chronic inflammatory diseases, such as CD, the strategies have not been applied to chronic pulmonary inflammation.

Interleukin-13 (IL-13) as a mediator may be implicated in COPD progression, and inhibition of IL-13 prevents the pathogenesis of COPD (Maneechotesuwan et al., 2016). During bacterial or virus infection of the respiratory tract, natural killer cells (NK cells) and DCs of lung tissue secrete IL-13 to activate the signal transducer and activator of transcription (STAT) 6 pathway, which leads to goblet cell hyperplasia, smooth muscle hyper-responsiveness, and airway remodeling (Horvat et al., 2010; Pritchard et al., 2016). IL-13 also involves in the pathogenesis of UC, but apparently not in CD. In UC patients, IL-13 secreted by invariant NK cells increases epithelial barrier dysfunction and apoptosis, reduces barrier function, and increases the NK cell cytotoxic action on the epithelium (Keely et al., 2012). As in COPD, STAT6 is an important mediator for preventing IL-13-induced epithelium barrier dysfunction and apoptosis in patients with UC (Rosen et al., 2011). Therefore, the STAT6 pathway is a novel therapeutic target in patients with CODP and UC. Although the increase in the serum IL-13 concentration in COPD patients may drive the aberrant NKT cells and macrophage responses across organs, whether these pathways adapt the systemic responses in IBD is unclear.

### Potential Mechanisms of the Skin-gut Axis

**FIGURE 2:**
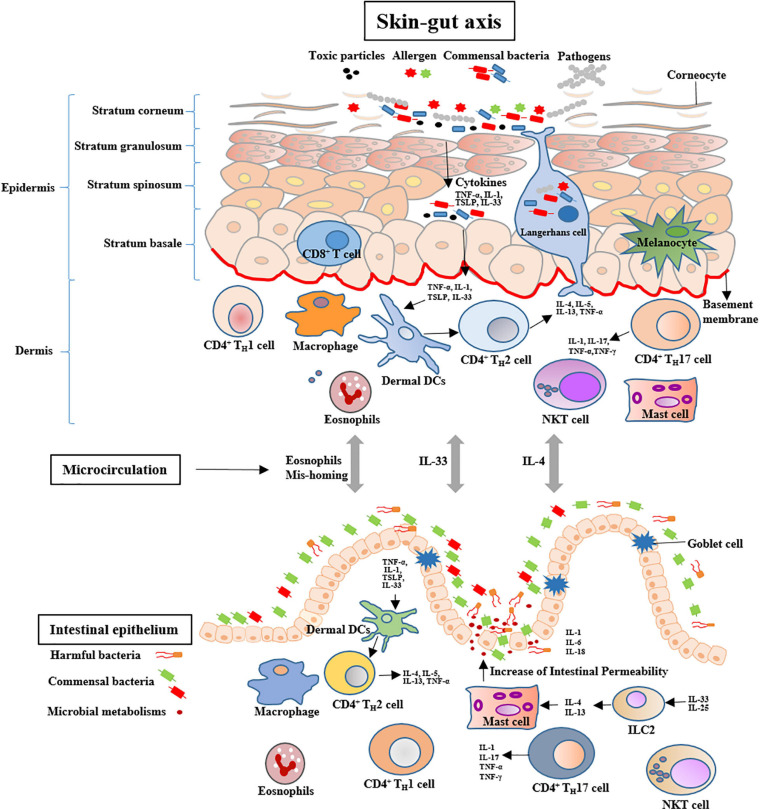
Potential mechanisms of skin-gut cross talk include increases in the number of intestinal MCs and skin-originating eosinophils mis-homing. Systemic IL-4 may activate the intestinal MCs to increase the permeability of the gut. Systemic IL-33 may increase the number of mucosal MCs in the small intestine via ILC2 activation. MCs = Mast cells; IL-4 = Interleukin-4; ILC2 = Type 2 innate lymphoid cells.

Skin is composed of epidermis and dermis, which exerts the important roles in maintaining water and electrolytes homeostasis and providing an immune barrier (van Splunter et al., 2020). Exposure to cigarette smoke not only contributes to premature skin aging and alopecia, but also induces various skin diseases, such as dermatitis, psoriasis, and skin cancer (Daling et al., 1992; Merimsky and Inbar, 1998). Cigarette smoking affects the skin health through different mechanisms, such as disturbing the balance of skin microbiome, promoting the pro-inflammatory cytokines, perturbing the balance in the protease/antiprotease systems, regulating the dermal microvasculature and degrading the skin collagen (Lahmann et al., 2001; Rossi et al., 2014). Moreover, the cutaneous manifestations implicate in other tissular and organic diseases, such as lung and intestinal diseases (Tromm et al., 2001; Ishigaki et al., 2013). Dysbiosis of intestinal microbiota closely associates with the skin diseases, such as acne, psoriasis and atopic dermatitis (van Splunter et al., 2020). Cigarette smoking might induce to intestinal microbiota dysbiosis through the skin-gut axis (Figure 2).

The skin-gut axis is a newly proposed concept that might be used to regulate intestinal homeostasis and health. Bosman et al. (2019) reported that the skin exposed to Narrow Band Ultraviolet B light to increase serum vitamin D levels can improve the relative abundance of the Lachnospiraceae. The incidence of CD in psoriatic are three folds more than that of healthy individuals (Gulliver, 2008). In a combination murine model of psoriatic dermatitis and colitis, the treatment of toll-like receptor 7 agonist imiquimod (IMQ) leaded to significantly decreased numbers of IgD + and IgM + B cells in the gut and abundances of *Lactobacillus johnsonii* and *Lactobacillus reuteri* in the intestinal tract (Kiyohara et al., 2019). These findings indicate that skin inflammation might contribute to the intestinal disorders through immunologic regulations and microbial community changes. Exposuring to allergen, thymic stromal lymphopoietin (TSLP) as a cytokine is heavily secreted by the epithelial cells of the skin, which increases naïve T cell differentiation toward an inflammatory phenotype through conditioning DC maturation. The complex of TSLP-DCs induces Th2 cells to produce IL-4, IL-5, IL-13, and TNF-α. Moreover, high TSLP production leads to activation and migration of Langerhans cells from the epidermis toward the dermis and an increase of activated DCs in the dermis (van Splunter et al., 2020). In addition, skin allergy induces systemic Th12 response, as noted by the increases of antigen-specific IgE, IL-4, IL-5, and IL-13 (De Benedetto et al., 2012). IL-4 targets mast cells (MCs) to expand in the intestine, which increases intestinal permeability and induces inflammatory response in sensitized mice (Leyva-Castillo et al., 2019). Moreover, IL-33 as an epithelial cytokine can be produced by fibroblasts, keratinocytes, epithelial and endothelial cells. The serum level of IL-33 in patients with atopic dermatitis was reported to be ten times more than that of control subjects (Tamagawa-Mineoka et al., 2014). Systemic IL-33 produced by keratinocytes synergizes with intestinal IL-25 to drive the expansion and activation of intestinal Type 2 innate lymphoid cells (ILC2s) that produces IL-4 and IL-13, which increases the number of mucosal MCs in the intestine (Leyva-Castillo et al., 2019). In an atopic dermatitis-like mouse model, IL-33 induces gastrointestinal allergy (Han et al., 2018). This suggests that IL-33 might exert an important role in the skin-gut cross talk. Furthermore, allergen exposured skin elicits the level of eosinophil of allergen unexposed mucosal organs and causes allergic inflammation, including the lungs and gut (Olbrich et al., 2020).

## Dysbiosis of Intestinal Microbiota and Systemic Disorders

**FIGURE 3:**
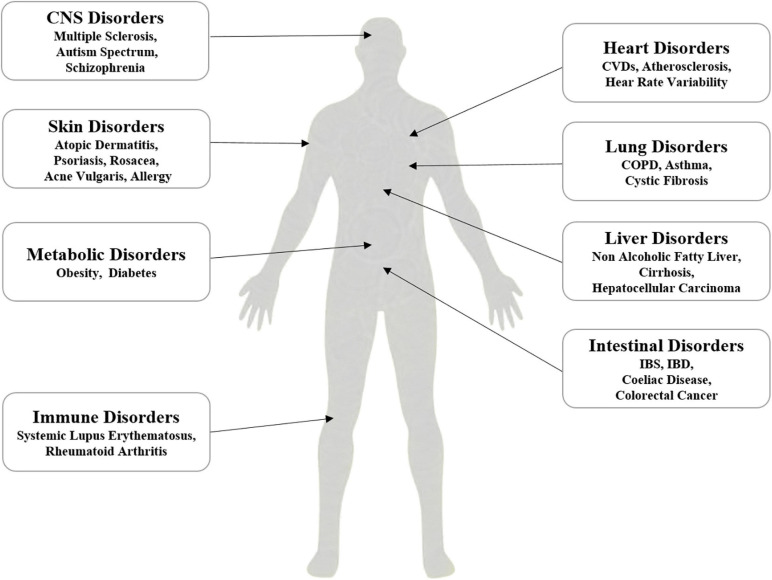
Proposed association of dysbiosis of intestinal microbiota with systemic disorders, which include the disorders related to liver, lung, heart, gut, skin, metabolic, immunity and CNS.

Intestinal microbiota exerts the key roles in the physiology of human being, and chronic cigarette smoking as an environmental factor induces dysbiosis of intestinal microbiota (Lee et al., 2018). Dysbiosis of intestinal microbiota associates with systemic disorders, such as intestinal disorders (Opstelten et al., 2016), metabolic disorders, central nervous system-related disorders (De Angelis et al., 2015; Tremlett et al., 2016), and autoimmune disorders (Hernán et al., 2001; Di Giuseppe et al., 2014; López et al., 2016; Figure 3).

### Dysbiosis and Intestinal Disorders

Intestinal microbiota exerts the important roles in intestinal homeostasis and diseases, and intestinal microbiota dysbiosis closely implicates in several gut-related diseases, such as IBS, IBD (Maslowski et al., 2009), and colorectal cancer (Hu et al., 2020). Compared with health controls, fewer species of the phylum Firmicutes were observed in IBD patients (Swidsinski et al., 2007). Moreover, there are significant difference in the intestinal microbiota of CD and UC patients (Swidsinski et al., 2002; Sokol et al., 2008). Cigarette smoking can worsen the development of CD, but it improves the symptoms of UC (Opstelten et al., 2016). In patients with IBS, the diversity and abundant of the overall gastrointestinal microbiota were significant difference from control subjects (Krogius-Kurikka et al., 2009). In addition, celiac disease and colorectal cancer associate closely with the dysbiosis of intestinal microbiota (Hu et al., 2020). However, it remains to be determined whether intestinal microbiota dysbiosis contributes to the pathology of intestinal diseases, or merely the result of a dynamic environment of intestinal tract. Compared with control subjects, germ-free mice exhibit the deficiency of immune system (Cebra, 1999). The increasing evidence supports that intestinal microbiota plays the important roles in the development and function of immune cells, such as T cells, NK cells, macrophages, dendritic cells, and invariant natural killer T (iNKT) cells (Liu et al., 2013). Moreover, administration of probiotics could effectively maintain the remission of several intestinal diseases, such as CD and UC (Shen et al., 2014).

### Dysbiosis and Skin Disorders

The bidirectional activity of gut-skin axis exerts the pivotal roles in the pathogenic mechanism of skin diseases. Intestinal microbiota is a major regulator of the gut-skin axis. Dysbiosis of intestinal microbiota closely associates with skin inflammation and diseases, such as atopic dermatitis, psoriasis, rosacea, and acne vulgaris (Szántó et al., 2019). A study assessed the presence of small intestinal bacterial overgrowth (SIBO) in patients with rosacea and found that the prevalence of SIBO in patients with rosacea was significantly higher than that of healthy individuals, whereas 46% patients with rosacea were cleared or markedly improved after oral administration of rifaximin (Parodi et al., 2008). Björkstén et al. (1999) reported that allergic children had less colonization with commensal *Lactobacilli*, *Bifidobacteria*, and *Bacteroides*, and greater colonization with aerobic bacteria. Moreover, mothers treated with probiotics pre- and post-natal significantly reduce the occurrence of atopic dermatitis in children (Kalliomäki et al., 2001). Breast milk provides various non-pathogenic bacteria that will colonize the infant’s intestine for developing microbiome. The antigens in breast milk have been processed by the mother’s gastrointestinal tract to reduce their allergenic potential. Probiotics supplementation can improve atopic dermatitis, which also supports the gut-skin axis (Zhu et al., 2018). The increasing evidence supports that cytokines and primed immune cells from the gut-associated Peyer’s patches might be transported to the skin through microcirculation, which regulates the immune status and destroys the epithelial barrier of the skin (Szántó et al., 2019).

### Dysbiosis and CNS-Related Disorders

In recent years, the interactions between the central nervous system and gastrointestinal tract have attracted much attentions, which increases the cognition of bidirectional communication between enteric nervous system and the brain (Cryan and Dinan, 2012). The evidence from animal studies supports that intestinal microbiota affects behavioral changes, especially in the stress response and stress-related behaviors (Nemani et al., 2015). Intestinal microbiota produces many neuroactive compounds, such as γ-aminobutyric acid, short-and long-chain fatty acids, and neurotransmitters, which affects the development and function of the CNS (Averina and Danilenko, 2017). Dysbiosis of intestinal microbiota has been implicated in many nervous systemic diseases, such as multiple sclerosis (Tremlett et al., 2016), autism spectrum (De Angelis et al., 2015), and schizophrenia (Nemani et al., 2015). Clinic studies reported that absence of the phylum Fusobacteria associates with relapse risk of multiple sclerosis compared with health individuals (Tremlett et al., 2016). The current evidence supports that multiple mechanism, such as neurocrine and endocrine pathways, appear to regulate the microbiota-gut-brain axis and brain in turn alter the composition of intestinal microbiota and behaviors through the autonomic nervous system. Currently, administration of antibiotics, fecal microbial transplantation and germ-free animal models are all being used to explore the effects of intestinal microbiota on the gut-brain axis (Mayer et al., 2015). However, it is unclear whether similar phenomenon would happen in healthy human or to disease states. Therefore, future works should focus on confirming that the microbiota-gut-brain also exists in healthy human or to disease states, such as IBD, IBS, and multiple sclerosis.

## Conclusion

Cigarette smoke toxicants perturb the dramatic balance of intestinal microbiota through various mechanisms. However, cigarette smoke is a complex mixture and the co-effect of multiple toxicants of cigarette smoke on the intestinal microbiota is yet unclear. Thus, a systematic investigation of the effect of cigarette smoke toxicants on the intestinal microbiome is greatly needed.

The lung–gut axis is a complex process involving multiple pathways and critical to our understanding of the etiology of complex inflammatory diseases in intestinal tract. Moreover, the skin-gut axis also contributes to the intestinal disorders. Our knowledge of these areas are mainly based on clinical observation, and basic research is needed to reveal those underlying mechanisms. Further understanding of the cross-links in gut and parenteral organs will enhance our knowledge of the physiological disturbances, which will be a major area for future research.

Intestinal microbiota dysbiosis is closely associated with intestinal and extra-intestinal diseases. Maintaining the balance of intestinal microbiota represents a new possibility for therapeutic approaches to smoking-related diseases. With advancement in techniques, multi-omics approaches will likely help to understand the intestinal microbiota dysbiosis and make human interventional studies with daily diet, exercise, and dietary supplements more meaningful.

## Author Contributions

All authors contributed to drafting the article and revising it critically for important intellectual content and contributed to the article and approved the submitted version.

## Conflict of Interest

The authors declare that the research was conducted in the absence of any commercial or financial relationships that could be construed as a potential conflict of interest.
